# Anti-Alzheimer and Antioxidant Effects of *Nelumbo nucifera* L. Alkaloids, Nuciferine and Norcoclaurine in Alloxan-Induced Diabetic Albino Rats

**DOI:** 10.3390/ph15101205

**Published:** 2022-09-28

**Authors:** Shahnaz Khan, Hidayat Ullah Khan, Farman Ali Khan, Afzal Shah, Abdul Wadood, Shujaat Ahmad, Mazen Almehmadi, Ahad Amer Alsaiari, Farid Ullah Shah, Naveed Kamran

**Affiliations:** 1 Department of Chemistry, University of Science and Technology, Bannu 28100, Khyber Pakhtunkhwa, Pakistan; 2 Department of Chemistry, Shaheed Benazir Bhutto University, Sheringal, Dir Upper 18000, Khyber Pakhtunkhwa, Pakistan; 3 Department of Biochemistry, Abdul Wali khan University, Mardan 23200, Khyber Pakhtunkhwa, Pakistan; 4 Department of Pharmacy, Shaheed Benazir Bhutto University, Sheringal, Dir Upper 18000, Khyber Pakhtunkhwa, Pakistan; 5 Department of Clinical Laboratory Sciences, College of Applied Medical Sciences, Taif University, P.O. Box 11099, Taif 21944, Saudi Arabia; 6 Department of Biochemistry, Rehman Medical Collage, Peshawar 25000, Khyber Pakhtunkhwa, Pakistan; 7 Lady Reading Hospital, Peshawar 25000, Khyber Pakhtunkhwa, Pakistan

**Keywords:** nuciferine, norcoclaurine, antidiabetic, anti-acetylcholinesterase, antioxidant enzymes effects, *Nelumbo nucifera* seeds

## Abstract

The present study is aimed to determine the efficacy and dose response of the nuciferine (**1**), norcoclaurine (**2**) and crude extract of *Nelumbo nucifera* in managements of diabetes, Alzheimer disease and related allergies. Experimentally, alloxan (100 mg/kg body weight (b.w.))-induced diabetic rats (200–250 g) were divided into seven groups (n = 6). Group I: normal control, Group II: diabetic control, Group III: standard treated with glibenclamide and Group lV-VII: treated with methanolic crude extracts (100, 200 mg/kg), nuciferine and norcoclaurine (10 mg/kg b.w.) for 15 days. Different tests were performed, including blood glucose, body weights and antioxidant enzyme assays, i.e., superoxide dismutase (SOD), catalase test (CAT), lipid peroxidation assay (TBARS), glutathione assay (GSH) and acetylcholinesterase (AChE) assay. Nuciferine and norcoclaurine significantly reduced blood glucose (*p* < 0.05) and restored body weight in diabetic rats. Moreover, nuciferine and norcoclaurine (10 mg/kg) significantly recovered the antioxidant enzymes (SOD, CAT, GPx and GSH) which decreased during induced diabetes. Significant increase in TBARS was also observed in the diabetic group and nuciferine as well as norcoclaurine (10 mg/kg) inhibited the increase in TBARS in diabetic animals (*p* < 0.05), as compared to glibenclamide. AChE activity was significantly recovered by nuciferine and norcoclaurine (10 mg/kg) both in the blood and brain of the diabetic group (*p* < 0.05). Nuciferine and norcoclaurine showed potent inhibitory effects against α-glucosidase and α-amylase with IC_50_, 19.06 ± 0.03, 15.03 ± 0.09 μM and 24.07 ± 0.05, 18.04 ± 0.021 μM, as confirmed by molecular docking studies. This study concludes that nuciferine and norcoclaurine significantly improve memory and could be considered as an effective phytomedicine for diabetes, Alzheimer’s disease (AD) and oxidative stress.

## 1. Introduction

Diabetes mellitus (DM) has been one of the deadliest causes of mortality in the world and at its prevalence (150 million world population) frequency, according to World Health Organization (WHO), will double by the year 2025. The problem with the use of modern drugs in the treatment of diabetes is not only their side effects, but their generally unattainable cost, especially for people in developing countries, is a major constraint. In diabetes physiotherapy, about 1200 medicinal plants are used universally and have proven to exhibit antihyperglycemic activities [1].

Reactive oxygen species and increased amounts of free radicals have been considered responsible for various prolonged and chronic diabetic problems especially in Type-1 and Type-2 diabetes. This causes it to affect the antioxidant defensive mechanism in the body, damage the cellular enzymes, organelles and cause diabetic complications [2].

Alzheimer’s disease (AD) is characterized by memory loss, cognitive dysfunction and instability in behavior, learning, planning and daily life activities [3]. The main pathological features are the occurrence of senile plaques, neurofibrillary tangles (NFTs) and neuronal damage in AD brains [4,5]. The extensive oxidative stress is a characteristic of AD brains in the accumulation of the traditional pathology of senile plaques and NFT [6]. The free radical damage increases and alters the expression or actions of antioxidant enzymes such as catalase and superoxide dismutase (SOD), which have been detected in both the peripheral tissues and central nervous systems of AD patients [7,8,9,10]. Furthermore, oxidative damage increases proteins and lipids in Mild Cognitive Impairment (MCI) and AD in brains. The degeneration of glutathione and antioxidant enzyme activities is further contained to the synapses and compared with the severity of the disease, indicates the involvement of oxidative stress in AD-related synaptic loss [11]. Type-2 diabetes mellitus (T2DM) and Alzheimer’s disease (AD) have shared abnormal levels of the enzymes: acetylcholinesterase (AChE) and butyrylcholinesterase (BuChE). Different levels of AChE and BuChE, both in AD as well as in T2DM, indicate that those two enzymes might be playing a fundamental part in the pathogenesis of the two disorders. T2DM and AD are both characterized by higher levels of AChE and BuChE in the plasma [12]. Similarly, the activities of both AchE and BuChE are found to be greater in diabetic patients against normal controls [13]. This shows that abnormal plasma levels of AchE and BuChE may act as indicators to predict the improvement of T2DM and AD in addition to serving as therapeutic targets [14]. The main objective of the present study was to determine anti-Alzheimer’s, and antioxidant effects of two isolated alkaloids, i.e., nuciferine and norcoclaurine as well as crude methanolic extract obtained from *N. nucifera* seeds in alloxan-induced diabetic albino rats. 

## 2. Results

### 2.1. Effect of Compounds and Crude on Blood Glucose and Body Weight

Table 1 illustrates the effect of various treatments of *N. nucifera*-derived samples; crude in 100–200 mg/kg body weight (b.w.)/day, nuciferine/norcoclaurine in 10 mg/kg body weight (b.w.)/day on blood glucose as well as on body weight of diabetic rats. The significant increase in blood glucose level was detected in the diabetic group (2.00 ± 1.32 mg/mL), as compared to the normal group (0.85 ± 1.53 mg/mL) and standard glibenclamide (0.94 ± 1.20 mg/mL) on the 15th day. The crude (1.11 ± 1.79 mg/mL) at 200 mg/kg, nuciferine (1.00 ± 1.45 mg/mL) and norcoclaurine (1.08 ± 3.15 mg/mL) at 10 mg/kg each significantly reduced the glucose level as compared to the diabetic group (*p* < 0.05). As a highly reactive substance, alloxan causes quick damage of β cells of langerhans, thus quickly increases the blood sugar and induces hyperglycemia [15]. The effect of nuciferine, norcoclaurine (10 mg/kg) and crude (100–200 mg/kg) on body weight has been shown in Table 2. The body weight of normal rats was detected to be stable (21 ± 1.43 g), whereas it declined in diabetic rats (149.87 ± 2.45 g). However, the diabetic group treated for 15 days with nuciferine (182.33 ± 3.05 g), norcoclaurine (178.91 ± 1.32 g) and crude at a concentration of 200 mg/kg (179.02 ± 2.71 g) significantly increased body weight (*p* < 0.05).

### 2.2. Antioxidant Enzymes Activity in Blood 

Table 3 shows a significant decrease in antioxidant enzyme levels, i.e., SOD, CAT and GSH, in diabetic groups. Furthermore, administrated with nuciferine, the SOD, CAT and GSH levels were significantly improved in diabetic groups as 7.1 ± 0.2/mg protein, 6.16 ± 0.7 E/min/mg protein and 86.96 ± 0.7/mg protein, respectively.

### 2.3. AChE Activity in Brain and Blood

The results for the AChE activity in different structures of brain homogenates are shown in Table 4. The AChE level was significantly raised in the diabetic group as compared to normal control (*p* < 0.05). The activity was significantly inhibited in the animals treated with nuciferine, norcoclaurine (10 mg/kg) and *N. nucifera* crude (100–200 mg/kg) in the cerebral cortex, hippocampus and striatum. Parallel results were also found in the cerebellum and hypothalamus (*p* < 0.05). Generally, oxidative stress is due to the formation of free radicals, tissues become spoiled and several diseases, such as cardiovascular, depression, joint and muscle, necrosis, diabetes and tumors, occur. Oxidative stress elevated in cognitive diseases, such as lipid peroxidation, has been reported in alloxan-induced diabetic rats, which finally results in prolonged diabetes [16]. Lipid peroxidation may happen due to the response of free radicals with lipids and increases the thiobarbituric acid reactive substances (TBARS), hydroperoxides and malondialdehyde [17].

Acetylcholinesterase (AChE) showed a significant role in mental abnormalities, memorial deficits and neurophysiological syndromes related with diabetes [18]. The current result reported a significant increase in AChE activity in several brain structures of diabetic rats including the cerebral cortex, cerebellum, hippocampus, hypothalamus and striatum, while the reduction in its activity was detected in the striatum and hypothalamus. 

Table 5 illustrates that the AChE activity was significantly increased in the blood of the diabetic group as compared with control (*p* < 0.05). While after 15 days of treatment, nuciferine, norcoclaurine (10 mg/kg) and *N. nucifera* crude methanolic extract (100–200 mg/kg) significantly inhibited the uncontrolled increase in AChE activity in the blood of diabetic rats, confirming that nuciferine, norcoclaurine and *N. nucifera* crude methanolic extract significantly improves memory in diabetic animals.

### 2.4. In Vitro α -Glucosidase and α-Amylase Inhibitory Effect

Nuciferine and norcoclaurine showed potent inhibitory effects against α-glucosidase with IC_50_ of 19.06 ± 0.03 and 15.03 ± 0.09 μM (standard IC_50_ = 12.02 ± 0.019), while both the compounds inhibited α-amylase with IC_50_ of 24.07± 0.05, 18.04 ± 0.021 μM (standard IC_50_ = 18.02 ± 0.11 μM), respectively. Norcoclaurine was revealed to be a competitive inhibitor against both the enzymes with outstanding effect almost on the same concentration as the standard drug (Table 6).

### 2.5. Molecular Docking 

**Figure 1:**
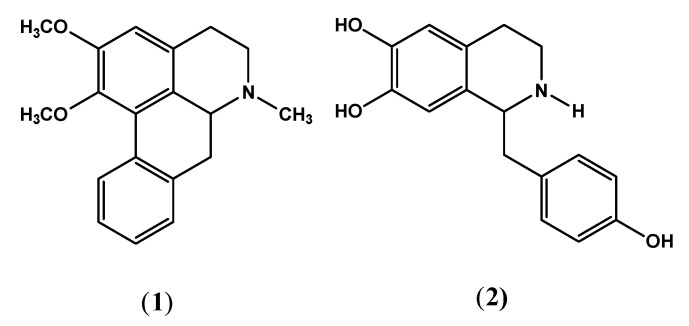
Nuciferine (**1**) and norcoclaurine (**2**) isolated from the seeds of *N. nucifera*.

**Figure 2:**
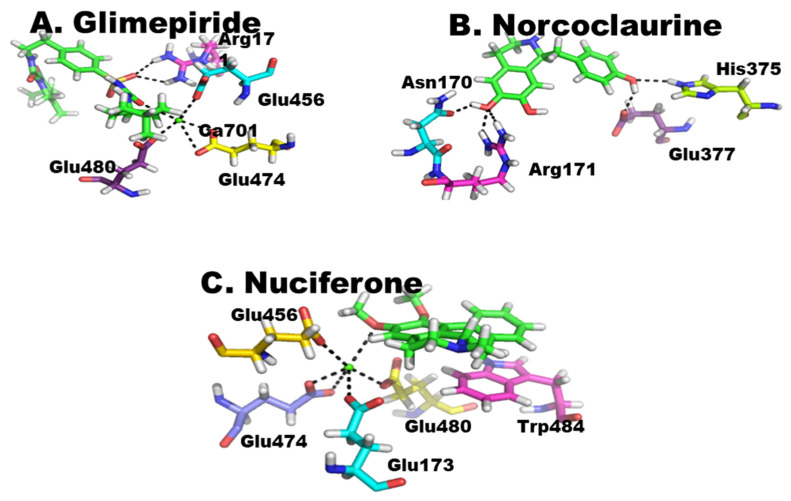
(**A**–**C**) Protein–ligand interaction of α-glycosidase. The green color shows the ligand.

**Figure 3:**
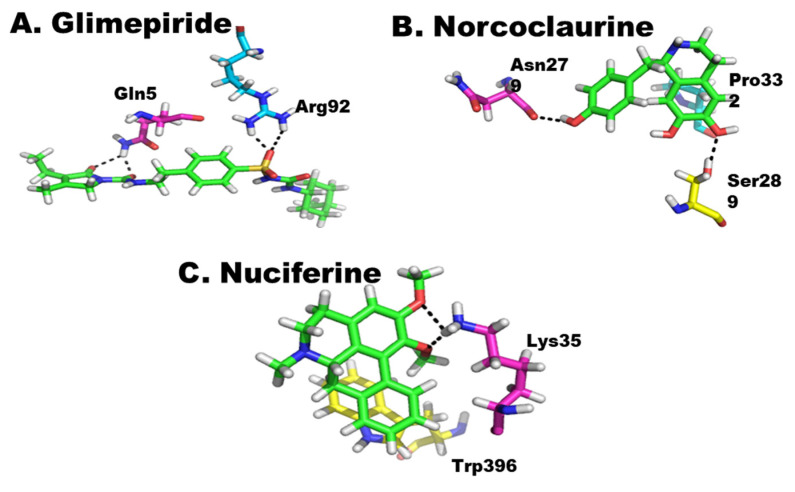
(**A**–**C**) Protein–ligand interaction of α-amylase. The green color shows the ligand.

Nuciferine (1) and norcoclaurine (2) (Figure 1) isolated from the seeds of *N. nucifera* along with the standard; glimepiride was computationally docked into the active site of α-glycosidase and α-amylase to establish their potential inhibition of both the enzymes. All three compounds showed strong interactions with the active sites of these enzymes and have the ability to affect the activities of these enzymes. Among these compounds, similar to in vitro studies, glimepiride was found to be most effective against both enzymes, followed by norcoclaurine and then nuciferine (Figure 2 and Figure 3). 

While assessing the virtual enzyme–ligand interactions, molecular docking against α-glycosidase, the standard drug, glimepiride forms two strong intermolecular hydrogen bonding with active site residue Arg171, as well as an intermolecular metallic interaction with Ca ion 701 through its electronegative OH group, which in turn binds to several active site residues such as Glu456, Glu474 and Glu480. Binding of glutamine residues in active site to Ca ion may be due to the attachment of its electronegative OH group to this electropositive ion (Figure 2A). Among the isolated compounds, norcoclaurine was found to be the most reactive by establishing strong interactions with active site residues of α-glycosidase. The compound formed five strong hydrogen bonds with active site residues of enzyme. Among three OH functional groups in norcoclaurine, two were found in interaction with enzyme residues at active sites. The hydroxyl oxygen of isoquinoline moiety formed an intermolecular bond with Asn170 as well as similar bond with Arg171, while oxygen of phenolic OH formed a molecular bond with Glu377. Besides these, the carbon at position 15 forms an H–pi interaction with six-ring of Trp484, as shown in Figure 2B. The other compound, i.e., nuciferine can also not be under estimated because it also has a considerable interaction. The six-ring of nuciferine has pi–pi interaction with five-ring of Trp484. Similarly, it has also intermetallic interaction with Ca ion at position 701 which in turn binds to a number of residues such as Glu456, Glu173, Glu474 and Glu480, as shown in Figure 2C. 

Similar to α-glycosidase, these compounds have strong interactions with α-amylase. The glimepiride has formed four intermolecular bonds with the active site residues of this enzyme. The oxygen of compound at position 30 forms two hydrogen bonds with the amino groups of Arg92, and the oxygen at position 50 and at position 57 formed hydrogen bonds with the nitrogen of Gln5, forming a stable protein–ligand complex. Protein–ligand interaction has been shown in Figure 3A. The norcoclaurine forms stable chemistry with the enzyme, forming three intermolecular interactions with active site residues. The two oxygens of the compound at positions 31 and 36 formed hydrogen bonds with the OH groups of Asn279 and Ser289, respectively, while six-ring of the compound formed pi–H interaction with Pro332, as shown in Figure 3B. Similarly, nuciferine also has considerable interaction with the active site of enzyme. The oxygen at position 39 forms a bond with Lys35 and its six-ring formed pi–pi interaction with the five-ring of Trp396, as shown in Figure 3C.

## 3. Discussion

This study showed that nuciferine and norcoclaurine have effective parts in falling glucose levels as well as in improving body weight, significantly reducing the fasting glucose level of blood in alloxan-induced diabetic rats as compared to the normal control. The decline in the body weight observed in the diabetic control group was also stated to be improved [19,20]. It was observed that nuciferine, norcoclaurine and *N. nucifera* methanolic extract strongly inhibited the lipid peroxidation level in the diabetic rats and thus, preventing oxidative damage of tissues, organs and lipid peroxidation in diabetes. Furthermore, highly reactive oxygen species (ROS) can decline the performance of the resistance system of antioxidant enzymes. CAT, SOD, and GPx are well-known protective enzymes because they have a potent role in the scavenging of free radicals in tissues and cells [21,22]. The scavenging ability of antioxidant enzymes observed to decrease in diabetic rats was successfully changed to close to normal in diabetic animals treated with glibenclamide group III as well as our sample groups IV, V, VI and VII. The absence of regularity of AChE activity may be due to efficient heterogeneity in the central nervous system. The cholinergic neurons may be varying in different regions. For example, the striatum and hypothalamus have fewer existing cholinergic neurons and low AChE activities related to other brain structures [23,24]. The parallel results of AChE activity in the serum and cerebral cortex of diabetic rats have also been observed by other studies [25]. The significant reduction in AChE activity in the brain might be due to decline in the transcription and translation of genes as well as enhanced cholinergic activity, which recovers cognitive function [26]. Oxidative stress and antioxidant system shows a vital role in pathophysiological cerebral modifications. Both SOD and CAT have significant role in the defense against oxidative stress. They decrease hydrogen peroxide and stop the generation of hydroxyl radicals, thus protecting cellular elements from oxidative loss [27]. The diminishing in the glycemic mechanism causes inhibition of neuronal activity. Cerebral extract from alloxan diabetic rats significantly inhibited the brain AChE activity of normal animals, indicating the presence of an inhibiting feature in the cerebrum of diabetic rats [18]. Cholinesterases (AChE and BuChE) are essential enzymes associated to memorial and cognitive functions, and determined hyperglycemia may activate memory loss, mainly in Type-2 diabetes mellitus [28]. Similarly, AChE and BuChE are important enzymes in the controlling of neurodegenerative diseases, such as Alzheimer’s disease, a new additional secondary complication of diabetes mellitus [29].

## 4. Materials and Methods

### 4.1. Plant Materials

Seeds of *Nelumbo nucifera* (fully dried and matured) were purchased locally from public market after identification by plant taxonomist and Professor Abdur Rehman, Government Post Graduate College, Bannu, KP, Pakistan. A voucher specimen (accession number BG-201) was submitted at the herbarium of University of Science and Technology, Bannu.

### 4.2. Extraction and Isolation of Compounds

Acid–base extraction method was used in the extraction of alkaloidal fraction from the seeds of *N. nucifera*. Briefly, 8 kg of seeds was ground to fine powder and soaked in methanol for seven days (cold maceration). The extract was filtered and concentrated on a rotary evaporator to yield gummy residue (440 g). This methanolic crude was pooled with 4 L of acidic-aqueous solution (pH 1.5, 0.5 N H_2_SO_4_) and stirred for about 1 h. The suspension was filtered and extracted with chloroform (3 × 2 L) in separating funnel to remove other non-soluble components. The acidic-aqueous portion was then basified with 10% KOH (pH 8–10, 4 L), and the resulted suspension was again extracted with chloroform to obtain the free alkaloids. This chloroform fraction upon concentration in vacuum yielded Total Alkaloids (TA) mixture (15 g). TA was subjected to chromatographic separations using various techniques. Open column chromatography using silica gel (70–230, GF_254_) and elution with increasing polarities of *n*-hexane, acetone and methanol (gradient manner) afforded various sub-fractions (NA1-NA9). The same solvent system in gradient manner was applied for further purification of sub-fractions through micro column chromatography with the addition of diethylamine (2 drops). The fraction NA2 obtained from *n*-hexane: acetone: DEA (9:1:2 drops) furnished compound 1 (nuciferine) as white crystals (240 mg, m.p. 164–165 °C). The fraction NA7 obtained from *n*-hexane: acetone: DEA (2:8:2 drops) showed various single spots on TLC and was further chromatographed over preparative TLC with CHCl_3_: MeOH (8:2) and 2 drops of glacial acetic acid which yielded compound 2 (norcoclaurine) as white flaks (70 mg, m.p. 243–244 °C) (Figure 1). The compounds were identified and characterized through various physical and spectroscopic techniques and comparison with the literature (already been published) [30,31,32].

### 4.3. Chemicals

The reagents and solvents such as alloxan monohydrate, glucose, DTNB, sodium citrate buffer, ascorbic acid, EDTA, phenazine methosulphate and glibenclamide were purchased from Sigma (Hamburg, Germany). Methanol and other solvents used were of analytical grade while glucometer used was from Roche Diagnostics Corporation (Indianapolis, IN, USA).

### 4.4. Animals

Forty-two (42) fully matured albino rats (200–250 g b.w.) of Sprague–Dawley types were purchased from National Institute of Health, Islamabad, Pakistan and kept under normal conditions for 15 days with 12-h dark/light rotation.

### 4.5. Experimental Induction of Diabetes

Alloxan monohydrate injection (100 mg/kg) was applied to experimental rats to induce diabetic conditions followed by giving 5% glucose water for 28 h in order to decrease the death probability in alloxan-induced rates from hyperglycemic shock. Over 300 mg/dL (3 mg/mL) blood glucose level (fasting blood plasma) was considered diabetic and a selective parameter for further study [33].

### 4.6. Experimental Design

The rats were divided into seven groups (six rats in each group). Normal and diabetic control groups I and II, standard glibenclamide (10 mg/kg) group III, *N. nucifera* crude (100–200 mg/kg) groups IV and V and nuciferine and norcoclaurine (10 mg/kg) group VI and VII. Rats were anesthetized after 15 days and their organ sections were collected for different activities. Blood was collected from each rat and centrifuged at 4000 rpm for 10 min for antioxidant enzyme activities. The brain was homogenized in 100 mM Tris buffer (Sigma, Hamburg, Germany) (pH 8.0). Protein variation for several brain structures was measured followed by protein determination of striatum (0.3 mg/mL), hippocampus (0.6.0 mg/mL), cerebellum (0.7 mg/mL) and hypothalamus (0.4 mg/mL and cerebral cortex (0.8.0 mg/mL)).

### 4.7. In Vivo Assessment

#### 4.7.1. AChE Calculation in Blood and Brain

In the brain (hippocampus, cerebellum, hypothalamus, striatum and cerebral cortex) and in the blood, AChE activity was measured using calorimetric method with slight modification [34]. The sample of 0.1 mL brain homogenate as well as blood serum (containing 100 mM potassium phosphate buffer (pH 7.5), 1 mM DTNB and 0.8 mM acetylcholine iodide (Sigma, Hamburg, Germany) were tested as AChE enzyme. The increase in enzyme activity was measured at 412 nm after the formation of the yellow-colored anion of 4, 4-ditheo-bis-nitrobenzoic acid and experiments were performed in triplicates.

#### 4.7.2. Determination of Lipid Peroxidation Assay (TBARS)

The lipid peroxidation activity in the blood sample was determined by slight modification in Sohal’s method [35]. The reaction mixtures containing homogenate sample (0.2 mL) in 0.1 M phosphate buffer pH 7.4 (0.58 mL), ferric chloride (0.02 mL) and ascorbic acid (0.2 mL). The reaction mixture was incubated at 73 °C for 1 h and stopped the reaction by adding 10% trichloro-acetic acid. The reaction mixture was continuously boiled, shaken for 20 min in a water bath and centrifuged for 10 min. The formation of TBARS was monitored at wavelength of 535 nm.

#### 4.7.3. Catalase Assay (CAT)

Catalase activity was determined in blood samples by modified method of Chance and Maehly [36]. The assay mixture comprised 0.1 mL blood serum, 2.5 mL phosphate buffer (pH 5) and 0.4 mL H_2_O_2_ while the absorbance was measured at 240 nm.

#### 4.7.4. Superoxide Dismutase Assay (SOD)

Superoxide dismutase activity was determined by an assay mixture containing 0.3 mL blood sample, 1.2 mL sodium pyrophosphate, 0.1 mL phenazine methosulphate and 0.2 mL NADH. The reaction was stopped within a minute by adding 1 mL of glacial acetic acid and the concentration of product was monitored at 560 nm [37].

#### 4.7.5. Glutathione Peroxidase Assay (GSH-Px)

Glutathione peroxidase assay was carried out by reaction mixtures of 0.1 mL blood serum with 1.49 mL phosphate buffer (pH 7.4), 0.1 mL EDTA, 0.1 mL sodium azide, 0.1 mL NADPH, 0.05 mL GSH and 0.01 mL H_2_O_2_. The intensity of color loss of NADPH was measured at 340 nm [38].

#### 4.7.6. Reduced Glutathione Assay (GSH)

GSH assay was carried out by mixing the blood serum with substrate DTNB by the modified method of Biessels [39].

#### 4.7.7. In vitro α-Amylase and α-Glycosidase Assays

The α-amylase assay was carried by taking 0.1 g of potato starch in 100 mL of sodium acetate buffer to obtain starch solution (0.1% *w*/*v*). A total of 27.5 mg of α–amylase was dissolved in 100 mL distilled water to prepare enzyme solution. A total of 96 mM of 3,5 di-nitro salicylic acid solution was prepared and mixed with sodium potassium tartrate to obtain colorimetric reagent. Plant extract and compounds were mixed with starch solution. With the addition of α–amylase, the mixture was left for some time at 25 °C. Measurement was taken after 3 min. The maltose generation was calculated from production of 3-amino-5- nitro salicylic acid which was formed from the reduction of 3,5-dinitro salicylic acid. The absorbance was measured at 540 nm using a UV/visible spectrophotometer (BMS, New York City, NY, USA), while the percentage inhibition was measured from the absorbance (*A*) of sample and control though the following equation.

(1) $$\%\;\mathrm{Inhibition}=\frac{\left[\Delta A_{540}^{control}-∆A_{540}^{sample}\right]}{∆A_{540}^{control}}\times 100$$

The inhibitory activity of alpha glucosidase was performed by taking starch solution as a substrate (2% *w*/*v*). The starch used in this assay was sucrose. A 0.2 M Tris buffer (pH 8.0) was prepared. Plant extracts and compounds (200 µg/mL–800 µg/mL), starch solution and Tris buffer were incubated at 37 °C for 5 min. A total of 5 mM *p*-nitrophenyl-α-D-glucopyranoside solution (50 µL) in 0.1 M phosphate buffer (pH 6.9) was added to each well at different time intervals. Afterwards, the reaction was initiated by adding 1 mL of α-glucosidase (1 U/mL) to each well and the mixture was incubated for 40 min at 35 °C. A total of 2 mL of 6 N HCl was added to the solution to stop the reaction. The color intensity was measured at 405 nm with the help of spectrophotometer (BMS, USA) [40]. The enzyme inhibition potential was expressed as inhibition % calculated by using the following formula.

(2) $$\%\;\mathrm{Inhibition}=\frac{\left[∆A_{405}^{control}-∆A_{405}^{sample}\right]}{∆A_{405}^{control}}\times 100$$

### 4.8. Molecular Docking Studies of α-Glycosidase and α-Amylase

The two compounds nuciferine (1) and norcoclaurine (2) isolated from the seeds of *N. nucifera* along with glimepiride were docked into the active site of α-glycosidase and α-amylase. The PDB structures of these enzymes were downloaded from protein data bank by their respective PDB codes, i.e., 5HQA and 1BVN, respectively. Next, the structural coordinates of both the enzymes were subjected to a Molecular Operating Environment software (MOE, Version 2020-09, Chemical Computing Group, Montreal, QC, Canada), package for preparation to obtain a minimum energy conformation of the enzymes for docking purposes. Finally, molecular docking was carried out for the optimized structures of receptors utilizing the default molecular docking standard protocol in MOE. The top-ranking docked complex based on the protein–ligand interaction (PLI) profile was chosen for exploration of the binding mode. Pymol was used for protein–ligand interaction and visualization.

### 4.9. Statistical Analysis

The data were evaluated statistically by Student’s *t*-test. *p* values less than 0.05 and 0.01 were taken as significant. Values are expressed as mean ± S.E.M.

## 5. Conclusions

Valuable conclusions from the current research exposed ameliorative effects of isolated alkaloids nuciferine, norcoclaurine and crude methanolic extracts from *N. nucifera* seeds. It is recommended that *N. nucifera* should be used as an effective antidiabetic, anti-acetylcholinesterase and antioxidant agent. This study may eventually lead to discovering more effective agents for the treatment of neurodegenerative syndromes and controlling of Alzheimer’s disease (AD). Hence, more research on this therapeutic plant is suggested to exploit its hidden medicinal importance. The results of this study validate the traditional use of this drug in the treatment of diabetes and oxidative stress. In the near future, nuciferine, norcoclaurine and crude methanolic extract of *N. nucifera* will be a potential source of drugs for the management of Type-2 diabetes mellitus, allergies and neurodegenerative syndromes. Further detailed studies may be carried out to identify the active principles responsible for the diabetic effect and to comprehend the precise mechanism of action of the *N. nucifera* ingredients.

## Figures and Tables

**Table 1:** Effect on blood glucose levels in diabetic rats by *N. nucifera* crude, nuciferine and norcoclaurine.

Sample Treatment	Blood Glucose Level (mg/mL)			
Days =	0	5	10	15
Normal control (Nc)	0.95 ± 1.65	0.93 ± 1.25	0.88 ± 0.12	0.85 ± 1.53
Diabetic control (Dc)	2.52 ± 2.85	2.31 ± 2.32	2.10 ± 1.68	2.00 ± 1.32
Dc+ glibencamide (10 mg/kg)	2.00 ± 1.85	1.54 ± 1.68	1.37 ± 1.20	0.94 ± 1.20 *
Dc+ crude (100 mg/kg)	2.10 ± 2.85	1.65 ± 1.08	1.44 ± 1.03	1.20 ± 1.90
Dc + crude (200 mg/kg)	2.15 ± 1.35	1.60 ± 1.55	1.52 ± 0.97	1.11 ± 1.79 *
Dc + nuciferine (10 mg/kg)	2.05 ± 3.85	1.64 ± 2.51	1.42 ± 1.23	1.00 ± 1.45 *
Dc + norcoclaurine (10 mg/kg)	2.10 ± 4.67	1.70 ± 3.25	1.58 ± 1.43	1.08 ± 3.15

**Note:** Each value is mean ± SEM of six animals; * *p* < 0.05.

**Table 2:** Effect on body weights in diabetic rats by *N. nucifera* crude, nuciferine and norcoclaurine.

Sample Treatment	Variations in Body Weight (g)			
Days =	0	5	10	15
Normal control (Nc)	180.21 ±1.54	177.71 ± 1.21	178.21 ± 0.32	181.21 ± 1.43
Diabetic control (Dc)	170.12 ± 1.82	161.22 ± 1.51	154.32 ± 2.48	149.87 ± 2.45
Dc+ glibencamide (10 mg/kg)	180.11 ± 1.90	169.54 ± 2.41	171.23 ± 2.07	179.50 ± 1.40 *
Dc+ crude (100 mg/kg)	179.10 ± 2.81	161.22 ± 1.09	166.32 ± 1.04	171.63 ± 1.87
Dc + crude (200 mg/kg)	179.01 ± 2.67	163.43 ± 2.45	171.44 ± 0.87	175.02 ± 2.71 *
Dc + nuciferine (10 mg/kg)	177.43 ± 4.19	161.97 ± 2.21	173.22 ± 2.67	177.33 ± 3.05 *
Dc + norcoclaurine (10 mg/kg)	178.22 ± 7.61	159.85 ± 1.34	169.11 ± 3.01	178.91 ± 1.32 *

**Note:** Each value is mean ± SEM of six animals; * *p* < 0.05.

**Table 3:** Effect of *N. nucifera* crude, nuciferine and norcoclaurine on the TBARS and SOD, CAT, GPx and GSH enzymes activities in diabetic rats.

Parameter	TBARS (nmol MDA/mg Protein)	SOD SOD/mg Protein	CAT E/min/mg Protein	GPx nmol NADPH/min/mg Protein	GSH GSH/mg Protein
Normal control (Nc)	1.75 ± 0.5	7.31 ± 0.9	6.86 ± 3.8	8.12 ± 0.6	95.10 ± 5.04
Diabetic control (Dc)	3.10 ± 1.3 *	3.01 ± 0.3 *	2.91 ± 4.2 *	3.55 ± 0.9 **	45.30 ± 7.27 **
Dc + glibenclamide (10 mg/kg)	1.55 ± 0.8 *	5.91 ± 0.3 *	6.45 ± 3.9	7.80 ± 1.6 *	89.52 ± 4.74 *
Dc + crude (100 mg/kg)	2.65 ± 0.5	4.90 ± 0.2 *	2.32 ± 2.8 *	6.20 ± 2.8 *	67.15 ± 3.95
Dc +crude (200 mg/kg)	1.96 ± 0.7 *	5.20 ± 1.5 *	4.95 ± 3.5 *	7.10 ± 3.0 **	78.56 ± 4.19 **
Dc + nuciferine (10 mg/kg)	1.60 ± 0.8 *	7.1 ± 0.2 *	6.16 ± 0.7 *	7.98 ± 0.9 *	86.96 ± 0.7 *
Dc + norcoclaurine (10 mg/kg)	1.98 ± 0.9 *	6.2 ± 0.7 *	5.1 ± 0.3 *	6.70 ± 0.89 *	76.68 ± 0.4 *

**Note:** * *p* < 0.05: normal control vs. diabetic control; diabetic control vs. treated groups. ** *p* < 0.01.

**Table 4:** Effect of *N. nucifera* crude, nuciferine and norcoclaurine on AChE activity in various brain regions of diabetic rats (µmol AcSCh/h/mg of protein).

Sample	Cerebral Cortex	Cerebellum	Hypothalamus	Striatum	Hippocampus
Normal control (Nc)	53 ± 2.25	48 ± 2.15	45 ± 1.65	40 ± 1.55	40 ± 1.18
Diabetic control (Dc)	80 ± 5.12	75 ± 4.25	72 ± 4.05	70 ± 3.21	67 ± 3.91
Dc + glibenclamide (10 mg/kg)	55 ± 2.06 **	50 ± 1.20 **	50 ± 2.15 **	46 ± 1.59 **	43 ± 1.10 *
Dc + crude (100 mg/kg)	74 ± 2.15	66 ± 2.54 *	66 ± 2.62	69 ± 1.81	50 ± 2.01 *
Dc + crude (200 mg/kg)	65 ± 1.70 *	60 ± 2.50	61 ± 2.05 *	55 ± 1.92 *	47 ± 1.25 **
Dc+ nuciferine (10 mg/kg)	56 ± 1.41 **	53 ± 1.70 **	55 ± 3.20 **	50 ± 1.90 **	41 ± 1.10 *
Dc + norcoclaurine (10 mg/kg)	60 ± 0.80 *	57 ± 1.40 *	60 ± 4.30 *	56 ± 0.90 *	48 ± 0.20 *

**Note:** * *p* < 0.05: normal control vs. diabetic control; diabetic control vs. treated groups. ** *p* < 0.01. Crude (methanolic extract of *N. nucifera*).

**Table 5:** Effect of *N. nucifera* crude, nuciferine and norcoclaurine against AChE activity in blood of diabetic rats (µmol AcSCh/h/mg of protein).

Sample	AChE Inhibition (%)
Normal	40 ± 2.51
Diabetic control	75 ± 3.59
Diabetic + glibenclamide (10 mg/kg)	45 ± 2.01 *
Diabetic + crude (100 mg/kg)	56 ± 2.61 *
Diabetic + crude (200 mg/kg)	52 ± 2.55 *
Diabetic + Nuciferine (10 mg/kg)	42 ± 7.43 **
Diabetic + Norcoclaurine (10 mg/kg)	55 ± 1.23 *

**Note:** * *p* < 0.05: normal control vs. diabetic control; diabetic control vs. treated groups. ** *p* < 0.01. Crude (methanolic extract of *N. nucifera*).

**Table 6:** The α-Glucosidase and α-Amylase Inhibitory Activities of Nuciferine and Norcoclaurine (IC_50_, μM).

Sample	α-Glucosidase ± SEM	α-Amylase ± SEM	Type of Inhibition
Nuciferine	19.06 ± 0.03	24.07 ± 0.05	Non-competitive
Norcoclaurine	15.03 ± 0.09	18.04 ± 0.021	Competitive
Glimepiride	12.02 ± 0.019	18.02 ± 0.11	--

**Note:** Each value is mean ± SEM of three replicates.

## Data Availability

Data is contained within the article.

## Sample Availability

Samples of the compounds and extract are available from the authors.
